# Attentional cueing: Gaze is harder to override than arrows

**DOI:** 10.1371/journal.pone.0301136

**Published:** 2024-03-28

**Authors:** Inka Schmitz, Hanna Strauss, Ludwig Reinel, Wolfgang Einhäuser

**Affiliations:** Institute of Physics, Physics of Cognition Group, Chemnitz University of Technology, Chemnitz, Germany; University of Bologna, ITALY

## Abstract

Gaze is an important and potent social cue to direct others’ attention towards specific locations. However, in many situations, directional symbols, like arrows, fulfill a similar purpose. Motivated by the overarching question how artificial systems can effectively communicate directional information, we conducted two cueing experiments. In both experiments, participants were asked to identify peripheral targets appearing on the screen and respond to them as quickly as possible by a button press. Prior to the appearance of the target, a cue was presented in the center of the screen. In Experiment 1, cues were either faces or arrows that gazed or pointed in one direction, but were non-predictive of the target location. Consistent with earlier studies, we found a reaction time benefit for the side the arrow or the gaze was directed to. Extending beyond earlier research, we found that this effect was indistinguishable between the vertical and the horizontal axis and between faces and arrows. In Experiment 2, we used 100% “counter-predictive” cues; that is, the target always occurred on the side opposite to the direction of gaze or arrow. With cues without inherent directional meaning (color), we controlled for general learning effects. Despite the close quantitative match between non-predictive gaze and non-predictive arrow cues observed in Experiment 1, the reaction-time benefit for counter-predictive arrows over neutral cues is more robust than the corresponding benefit for counter-predictive gaze. This suggests that–if matched for efficacy towards their inherent direction–gaze cues are harder to override or reinterpret than arrows. This difference can be of practical relevance, for example, when designing cues in the context of human-machine interaction.

## Introduction

In human interactions, visual attention is often directed by another person’s gaze [1]. Therefore, gaze is an important social cue by which interaction partners guide each other’s attention to the same location or object, thereby establishing joint attention [2]. Gaze also plays an important role in human-machine interaction. In particular, interactions with embodied digital technologies, such as robots, can feel more socially realistic and come with an increased impression of mutual understanding when the artificial agent is capable of establishing joint attention [3]. The ability of the robot to respond to joint attention can enhance performance in a joint human-robot task [4]. Moreover, socially realistic shifts of gaze by a robot can lead human interaction partners to increase their acceptance of the robot, for example in small-talk situations [5]. To experimentally investigate the effect of human and artificial gaze on human attention allocation, spatial cueing paradigms are frequently used [6–8].

**Fig 1:**
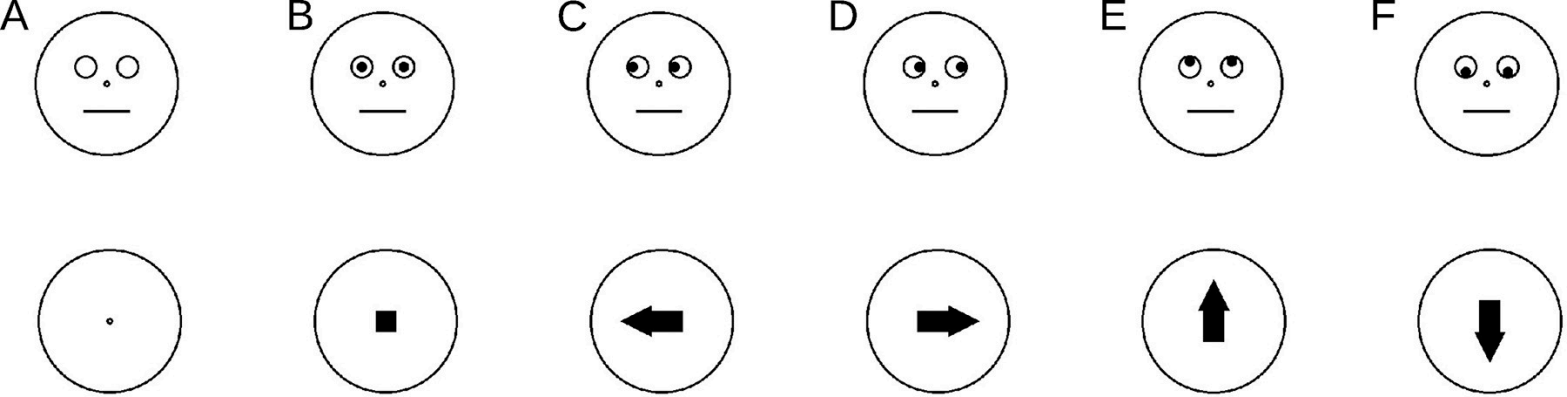
Exemplary subset of the used cueing stimuli. A) Initial placeholder, B-F) cues B) neutral, C) left, D) right, E) up, F) down. Stimuli are shown to scale, diameter of circles was 6.8 dva.

The foundational work on gaze-based attentional effects was done by Friesen and Kingstone [9] and has its roots in the cueing paradigm introduced by Posner in his seminal work on attentional effects of central cues [10]. Friesen and Kingstone presented schematic faces that could gaze towards the left or the right (Fig 1A–1D). Unlike in Posner cueing, where the cue indicates the probable location of the target, the gaze direction was not predictive of the subsequent target location. Instead, the target could appear on either side with equal probability and independent on the gaze direction. Nevertheless, Friesen and Kingstone found a reaction time advantage for target detection, localization and identification when the eyes of the face pointed in the direction of the target stimulus (the “cued” side) as compared to the gaze pointing in the other direction (the “uncued” side). In general, no reaction time disadvantage was found for the uncued condition. This result differs from Posner’s, who used arrow cues that–in contrast to Friesen & Kingstone’s faces–were predictive of the target location. After presenting an arrow cue, a target appeared in the arrow direction in 80% of cases (“valid cues”), and on the opposing side in 20% of cases (“invalid cues”), so that the cue direction was predictive for the target position. Responses to targets were faster for valid than for invalid cues. In addition, Posner used “neutral” cues, which were not predictive regarding the side of the target. Neutral cues led to reaction times in-between valid and invalid conditions, demonstrating the reallocation of resources to the valid side at the expense of the invalid side, in line with James’ classical characterization of attention [11]. In an earlier work of Posner, he found attentional costs in the predictive 80% probability condition but not in the non-predictive 50% probability condition of a matching task [12].

Interestingly, Posner chose arrows as cues, which in principle may confound two aspects [10]: the experimentally induced validity manipulation and the directional information inherent to the arrow symbol. That is, on top of the validity effect, there can be an effect of the arrows’ inherent meaning irrespective of their predictiveness, similar to Friesen & Kingstone’s face cues [9]. In recent decades, similarities and differences between gaze and arrow cues have been intensely studied (see [13] for a meta-analysis) adding to research that focuses on the gaze cue effect itself, which investigates features that may contribute to the reaction time advantage of cued vs. uncued locations. Gaze and arrow cues appear to act similarly in many respects–for example, both cannot be ignored [14]. The effect of gaze cues has been shown to be robust in a variety of studies, with the effect size depending on the type of task and on stimulus properties (see [15] for a review and [16] for a meta-analysis): for example, gaze cueing effects are modulated by facial expressions [17].

Research on gaze cues is often motivated by the question of uniqueness of gaze as a social cue compared to non-social cues: There might be specific social mechanisms, such as the "watching eyes effect" [18]. Even infants show a very simple form of gaze following, presumably based on a preference for faces in combination with motion cues [19] and it is plausible that prioritized processing of gaze has evolved as an evolutionary advantage, supported by the anatomy of the human eye with its white sclera. Even though gaze and arrows show largely similar attentional effects in spatial cueing paradigms, the remaining differences–e.g., regarding counter-predictive cueing [20]–are of high interest to increase the understanding of gaze as a social cue. In some cases, arrows and gaze can have apparently opposing effects [21]: for example, congruency between gaze direction and non-central presentation location quickens the response in case of arrows, but slows the response in case of gaze cues, presumably as a consequence of the distinct assumed reference frames. Reaction time advantages due to such non-predictive cues, suggest a reflexive attentional shift, i.e., an involuntary processing component that cannot be fully suppressed by participants in these experimental setups. Involuntary attentional orienting–for both gaze and arrows–can be modulated by the volitional use of cues, depending on individual differences [22].

The ongoing development of robots and other autonomous agents has led gaze-cueing research to obtain a particular focus on artificial gaze in addition to human gaze or gaze in general. Admoni and colleagues used different types of central cues (arrows, real faces, robots, schematic faces) in a cueing paradigm [6]. Here, the arrow and gaze direction were counter-predictive, i.e., the targets were more likely to appear on the opposite side of the indicated direction. Targets could appear on the vertical and horizontal axis, but one axis was never cued and the probability of appearance was also substantially lower compared to the other axis. The results show different attentional effects for robot and non-robot stimuli. In contrast to this study, Morillo-Mendez and colleagues showed a clear effect of robotic cues in an experiment using only non-predictive cues on the horizontal axis [7], as in [9]. By presenting head motion from the front perspective in addition to head motion from the back perspective, they in addition showed that eye visibility is not necessary to direct attention and that this effect does not rely on low-level motion cues. Such findings support the notion that artificial gaze displays can appropriately guide the visual attention of human interaction partners. Because artificial agents can have many different appearances, simple signs as arrows and schematic faces are still of interest for guiding visual attention with displays. In context of approaching behavior of robots, there are already studies on lights and arrows (e.g.,[23]), but also on eyes and gestures as nonverbal cues (e.g., [24]).

Since the human eye is typically elongated along the horizontal axis, natural gaze expression will usually differ between the horizontal and the vertical. This raises the question whether gaze cueing also shows differences between the vertical and the horizontal direction. Differences in cueing effects between these axes have been found in some conditions [25,26], although most of the cueing literature finds no evidence for a horizontal/vertical asymmetry in standard cuing paradigms (e.g., [10,27]). Such differences for gaze cues may be coincidental or caused by the design of the specific experiments. However, as there are axial asymmetries in gaze behavior and perception (e.g., of fixations in visual search, [28]; gaze estimation on photographs, [29]), we consider it important to assess whether there are differences between horizontal and vertical gaze cueing when stimuli and task themselves do not have such asymmetries. While both axes have been used for schematic faces before, a direct comparison is still lacking. In Experiment 1 of the present study, we tried and replicated one condition of Friesen & Kingstone’s gaze cuing paradigm [9]–their identification task at a stimulus onset asynchrony (SOA) of 300 ms. The particular SOA was chosen as it showed substantial effects in Friesen and Kingstone’s task and as gaze cueing effects at 300 ms SOA have already been observed in robots [30]. We further extended the replication to the vertical direction. Moreover, we compared the effect of gaze cues to visually closely matched arrow cues.

Having established with Experiment 1 a stimulus set and paradigm, in which the effects of non-predictive arrows and non-predictive faces were closely matched, we tested in Experiment 2, whether their effects remain similar when they become counter-predictive rather than non-predictive. We hypothesized that cueing effects of arrows (overtrained, but non-social cues) can more easily be overridden than the effect of faces (overtrained and social cues). This hypothesis is tested on overall cueing effects, the development of cueing effects over the course of the experiment and based on eye-tracking data. As an additional control condition, we introduced color cues, which had no inherent direction.

## Experiment 1

### Methods

#### Participants

16 participants (9 women; 7 men; *M =* 24.06 years, *SD =* 3.49 years) with normal or corrected-to-normal vision participated in Experiment 1 from January 12, 2023 to February 21, 2023. Each participant completed 4 training blocks of 18 trials each, and 8 experimental blocks of 90 trials each. After each block, the participants could take a break. Participants were recruited via a dedicated mailing list from the TU Chemnitz community and the experimenters’ circle of acquaintances. We determined the sample size based on an a priori power analysis for a within-subjects design, with balancing (order arrow/face × order vertical/horizontal × key assignment) requiring a sample size that is a multiple of 8. Assuming a strong effect (*f* = .4), a power (1-b) of 80%, and an alpha level of .05, this yields *N* > = 12 for a three-level factor and N > = 15 for a two-level factor. Written informed consent was obtained from all participants prior to participation. The local ethics committee of the TU Chemnitz reviewed and approved all procedures of both experiments, including the planning of the sample size (case no. #101549112).

#### Stimuli and setup

The stimuli were generated in MATLAB (Mathworks, Natick, MA) and presented using MATLAB with the Psychophysics Toolbox [31,32] on a VIEWPixx-Monitor (VPixx Technologies; size: 523 mm x 300 mm; resolution: 1920 px × 1080 px; refresh rate: 120 Hz; distance from observer: 57 cm). The experiment was conducted in a testing chamber, with no light source other than the monitor. We measured the gaze positions at a sampling rate of 1000 Hz using the “Eyelink-1000” camera/infrared-system (SR Research, Canada). To maintain a stable viewing position in front of the screen and eye-tracking camera, participants sat in a chair with their head resting in a chin and forehead rest. Participants’ responses were captured using bottom and right buttons of the 5-button RESPONSEPixx Button Box (VPixx Technologies).

Schematic faces and arrows were used as directional cues. The black (<0.5cd/m^2^) line drawings were presented in the screen center (Fig 1). The gray background had a luminance of 10cd/m^2^. We created the face stimuli and the targets to match the properties reported by Friesen & Kingstone [9]: Each face consisted of an outer circle with a diameter of 6.8 degrees visual angle (dva). In the center of this circle, and thus in the center of the monitor, was a small black-filled circle with a diameter of 0.2 dva (nose). The eyes were represented by two circles with a diameter of 1.0 dva, positioned 1.0 dva apart from the vertical axis and 0.8 dva above the horizontal axis. The pupils were depicted as black-filled circles with a diameter of 0.5 dva. They were positioned either in the center of the eye circles or touching the left, right, upper, or lower edge of these circles. The mouth was represented by a horizontal line 2.2 dva wide, positioned centrally 1.3 dva below the nose. The line width was 0.11 dva.

In addition to the stimuli used by [9], we designed closely matched arrow cues (Fig 1, bottom row). The arrows were also surrounded by a black circle with a diameter of 6.8 dva and a central black dot with the same proportions as the nose served as the fixation point. The neutral cue stimulus was presented as a central black-filled square with a side length of 1 dva. Black-filled arrows pointing either up, down, right, or left were used for the cued and uncued conditions. These arrows consisted of a black rectangle of 1 dva width and 1.5 dva length, to which a triangle with a height of 1.5 dva and baselength of 1.5 dva was connected. Thus, the total length of the arrow was 3 dva from tip to rear. The arrows were placed in the center of the circle and shifted by 0.5 dva in the direction of the arrow.

The letters “F” and “T” (height: 1.3 dva, width: 0.8 dva) were used as targets and presented 6.0 dva to the left, right, above or below screen center. The design of the letters and the locations (in the left and right case) were also as closely matched to [9] as possible.

#### Procedure

Before the first block, participants were instructed to look at the center of the screen during the trials. They were also informed that the cues would not provide any information about the target nor about its location.

The experiment consisted of 12 blocks (including training blocks). Each block began with a "button check": Participants had to press the buttons (down and right) corresponding to the target ("F" and "T") in order to check that they had remembered the assignment correctly. The eye tracker was then calibrated before data collection for the block began. For each of the four conditions (face-horizontal, face-vertical, arrow-horizontal, arrow-vertical), a training block of 18 trials was followed by two experimental blocks of 90 trials. The participants’ task was to respond as quickly as possible to the target by pressing the corresponding button. To avoid a larger button correspondence to one of the target axes, the down and the right button were used, and the index finger of the right hand was placed on the down button and the middle finger on the right button.

**Fig 2:**
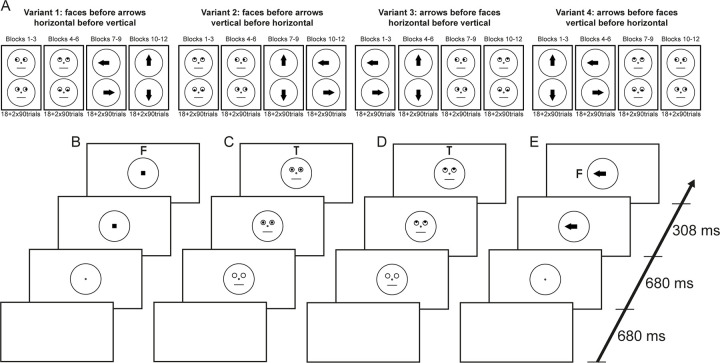
Procedure Experiment 1. A: The four orders of conditions that were used (counterbalanced across participants together with the button assignment to the targets), each condition consisted of one training block and two experimental blocks. B-E: Examples for trials (timeline on the right), B: neutral arrow trial with target on top, C: neutral face trial with target on top, D: face trial with valid cue to the top, E: arrow trial with valid cue to the right. The examples in B to E are not to scale (letters F and T and line width are scaled up for visibility).

In each block the targets appeared either on the horizontal axis or on the vertical axis. Blocks were ordered such that all six blocks of the same cue type (arrow, face) followed each other with either the first three vertical and the second three horizontal or vice versa. The three blocks (1 × training, 2 × experimental) of the same cue type (arrow, face) were presented consecutively. Per participant, the order of horizontal/vertical was identical for both cue types. This resulted in four possible orders of blocks (Fig 2A). The eight combinations of these four conditions with the two button-target assignments were counterbalanced across observers. In each block, one-third of the trials were neutral control trials, one-third were cued in the direction of the target, and one-third were cued in the opposite direction; the order of trials was random. Each target position, i.e., above or below and left or right of the cue, was presented the same number of times in each block.

The sequence within a trial followed closely Friesen and Kingstone’s paradigm. Each trial started with an empty gray screen. After 680 ms a face with no pupils (face conditions) or only the central black circle (arrow conditions) was presented (Fig 1A) and after another 680 ms the pupils or the arrows (the small square in the neutral arrow condition, Fig 1B–1F) appeared and remained visible until the end of the trial. Targets appeared 308 ms (300ms + 1 frame) after the cue onset and also remained until the end of the trial. See Fig 2 for an illustration of example stimulus presentation sequences.

#### Analyses

As a measure of accuracy, we computed the number of correct responses to the targets per individual, separately for the factors cue type (arrow, gaze), target orientation (i.e., the orientation of the axis along which targets could occur; horizontal, vertical) and cueing condition (cued, neutral, uncued). To statistically assess the effects of these factors, a 2 × 2 × 3 repeated-measures analysis of variance (rmANOVA) was performed.

In the analysis of reaction times (RTs) only correct trials of the experimental blocks were included. For each of these trials, we defined RT as the time from target onset to response button press. To analyze RT effects relative to the neutral condition, we defined cueing effects (CEs) as follows. The CE for the cued condition (CE_cued_) was defined as the difference between the median RT in the neutral condition (Mdn_neutral_) minus the median RT in the cued condition (Mdn_cued_). Equivalently, we defined the CE for the uncued condition (CE_uncued_) by subtracting the median RT of the uncued condition (Mdn_uncued_) from Mdn_neutal_. Both measures were computed separately by cue type (factor: cueType), target orientation (factor: tgtOrientation) and participant, but aggregated across blocks of the same condition and participant prior to taking the medians:

$$CE_{cued}(VP,\;cueType,\;tgtOrientation)=Mdn_{neutral}(VP,\;cueType,\;tgtOrientation)\;–Mdn_{cued}(VP,\;cueType,\;tgtOrientation)$$

$$CE_{uncued}(VP,\;cueType)\;=Mdn_{neutral}(VP,\;cueType,\;tgtOrientation)–Mdn_{uncued}(VP,\;cueType,\;tgtOrientation)$$

The sign of these differences was chosen such that a positive CE implies a faster response (smaller RT), and negative CE implies a slower response (larger RT) than in the neutral condition. To compare between conditions for our within-subject design, we used a 2 × 2 × 2 rmANOVA with the CE as dependent variable and factors cueType (levels: face, arrow), tgtOrientation (levels: horizontal, vertical) and cueing (levels: cued, uncued). For the analyses of the eye movements, we used the saccade detector of the Eyelink with saccade thresholds of 35°/s for velocity, and 9500°/s^2^ for acceleration.

### Results

#### Accuracy

On average, observers reached an accuracy of 95.8% correct responses. We did not find any dependence on the cue type (face vs. arrow; *F*(1, 15) = 2.07, *p* = .17, rmANOVA), target orientation (horizontal vs. vertical; *F*(1, 15) = 0.01, *p* = .92), or cueing condition (cued vs. uncued vs. neutral; *F*(2, 30) = 0.37, *p* = .70). Neither did we observe any significant interactions between these factors (all *p*s > .18). Observers responded faster in incorrect than in correct trials, with the mean over per-participant median RTs amounting to 405 ms (*SD* = 99 ms) and 443 ms (*SD* = 72 ms), respectively (*t*(15) = 4.38, *p* < .001, paired t-test).

#### Cueing effects

**Fig 3:**
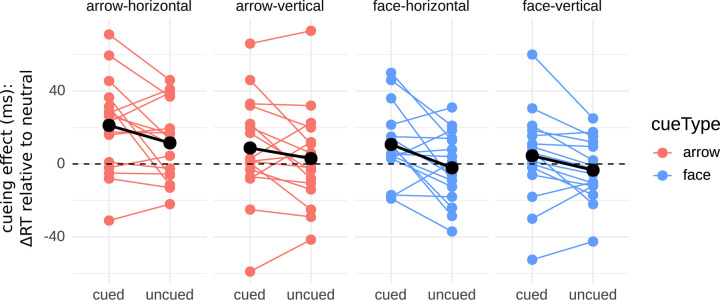
Cueing effects of Experiment 1. Red and Blue: Individual cueing effects (CE) based on medians of each of the 16 participants for each condition combination. Black: means of the CEs.

We analyzed whether cue type (face vs. arrow), target orientation (horizontal vs. vertical), and cueing condition (cued vs. uncued) affected the CE. We neither found a main effect of cue type, *F*(1,15) = 2.52, *p* = .13 (rmANOVA), nor a main effect of the target orientation, *F*(1, 15) = 1.54, *p* = .23. Furthermore, no interactions between any of the factors were observed (all *p*s > .30). However, there was a significant main effect of cueing, *F*(1, 15) = 10.87, *p* = .005, indicating that the cue had an effect on RT (Fig 3). Follow-up t-tests revealed a significant positive CE for the cued condition: *t*(15) = 3.14, *p* = 0.006, *M* = 11.28, *SD* = 14.37, but no CE (neither negative nor positive) in the uncued condition: *t*(15) = 0.87, *p* = 0.40, *M* = 2.37, *SD* = 10.45. This shows that there is a reaction time advantage for cues directed toward the target, but no reaction time disadvantage for cues directed in the opposite direction.

#### Eye tracking

An exploratory analysis of gaze positions from cue onset to target onset and during target presentation shows that in most trials participants followed the instruction to fixate the center of the screen during the trials, although we did not actively enforce this instruction: In the horizontal direction, saccades were made to positions less than 2 dva from the center in 26% of the trials and to positions greater than 2 dva in 12% of the trials. For the vertical axis, this was the case in 24% and 14% of the trials, respectively. We considered only saccades starting at least 100 ms after the cue onset, to minimize the proportion of saccades which are caused by incidents before the cue onset. No systematic deviation from the center or dependence on the cue direction was found.

## Experiment 2

Experiment 1 showed no differences between arrows and faces in their efficacy to cue towards a target despite being non-predictive. Since, unlike in [20], there was no evidence of differences between arrows and faces, we next asked whether the face as a social cue is harder to override than an arrow cue, if its predictiveness conflicts with their (over)trained (social) meaning. To this end, we examined the effect of counter-predictive cues. Counter-predictive cues point in the opposite direction of the targets in all trials–e.g., gaze down or an arrow pointing down is 100% predictive for a target that will appear *up*. Throughout Experiment 2 all arrow and face cues that were not neutral (Fig 1B) were counter-predictive. Based on the data of Experiment 1, we also made some further adjustments to the paradigm. Since there was no indication from Experiment 1 that differences between cue types depended on the respective axes, we restricted ourselves to the vertical direction; this choice simplifies the experimental design, as the task response (letter identification) can now be given by pressing a left or right button without potential congruency confounds to the up/down target location. The eye-tracking data in Experiment 1 showed that participants followed the instruction to fixate the center of the screen in most of the trials. Omitting this instruction would probably lead to more informative eye movements. It should be noted that this may also lead to a more overt attention allocation compared to the original paradigm. However, a more open instruction also provides the basis for more natural eye movements. Since comparability with exogenous cues as in [12] was not central in Experiment 2, this modification was adopted. In addition, we used color cues, which–unlike faces and arrows–have no social or overtrained inherent direction. The color condition was used for an initial training block and served as a filler to separate blocks with arrows from face blocks and was used to control for general changes in the CE over the course of the experiment.

### Methods

The overall setup, procedure and analyses of Experiment 2 were analogous to Experiment 1. The main changes were, that the face and arrow cues were directed to the opposite direction of the target position in 100% of the trials (i.e., they were 100% valid in Posner’s sense, but against their intuitive or overtrained meaning). Moreover, cues and targets appeared only along the vertical axis, and we used colors as an additional cueing condition. Additionally, there was no instruction to look at the center of the screen during trials to obtain more informative eye movements. Unless otherwise stated, recruitment, apparatus, procedures, analyses, and stimuli were identical to those used in Experiment 1.

#### Participants

For Experiment 2, we initially recruited the same number of participants as for Experiment 1 (*N* = 16; 13 women; 3 men; *M =* 21.88 years, *SD =* 3.12 years) to match Experiment 1 as closely as possible in this respect. After realizing that we would only be able to detect strong effects (*f* = .4, cf. the power analysis for Experiment 1) with this sample size, whereas a meta-analysis [13] found medium effect sizes for similar studies, we decided to replicate Experiment 2 with a larger sample. We based the power calculation for this additional experiment on the empirical effect size observed for the initial *N* = 16 sample. For the additional experiment, we consequently recruited *N* = 24 participants (17 women; 6 men; 1 undisclosed gender; *M =* 21.17 years, *SD =* 3.07 years). For the analyses planned *a priori*, we treated these two samples separately, referred to as Experiment 2a (*N* = 16) and Experiment 2b (*N* = 24 [prior to excluding one participant, see below]), respectively. For additional (exploratory) analyses, we aggregated the samples and refer to the joint sample as Experiment 2 (*N* = 40). The sample size of *N* = 40 would allow to detect an effect of *f* = 0.23 –i.e., a medium-size effect–under the original constraints (1-b = .8, a = .05).

The data collection period for Experiment 2a was from June 12, 2023 to July 4, 2023, for Experiment 2b from November 13, 2023 to November 30, 2023. Each participant completed one training block of 18 trials and seven experimental blocks of 90 trials each.

#### Stimuli and setup

In Experiment 2 the stimuli of the vertical presentation axis of Experiment 1 were used. As an additional condition, colored filled circles (cyan or magenta) of the same size as the other stimuli (6.8 dva diameter) were used. As neutral stimulus for the color blocks, the circle was filled with the background gray of the same luminance (10cd/m^2^) as the colors. In the center of the colored/gray circle was a square as it was also used in the arrow condition (Fig 1B, bottom).

#### Procedure

The experiment consisted of 8 blocks. For each participant, the first block was a training block of the color condition, and the second, fifth, and eighth blocks were experimental blocks of the color condition. Prior to the start of the experiment, participants were informed about the counter-predictive nature of the arrow and gaze cues and in which direction the targets would appear after a cyan or a magenta cue. This assignment of color to direction was counterbalanced across the participants.

**Fig 4:**
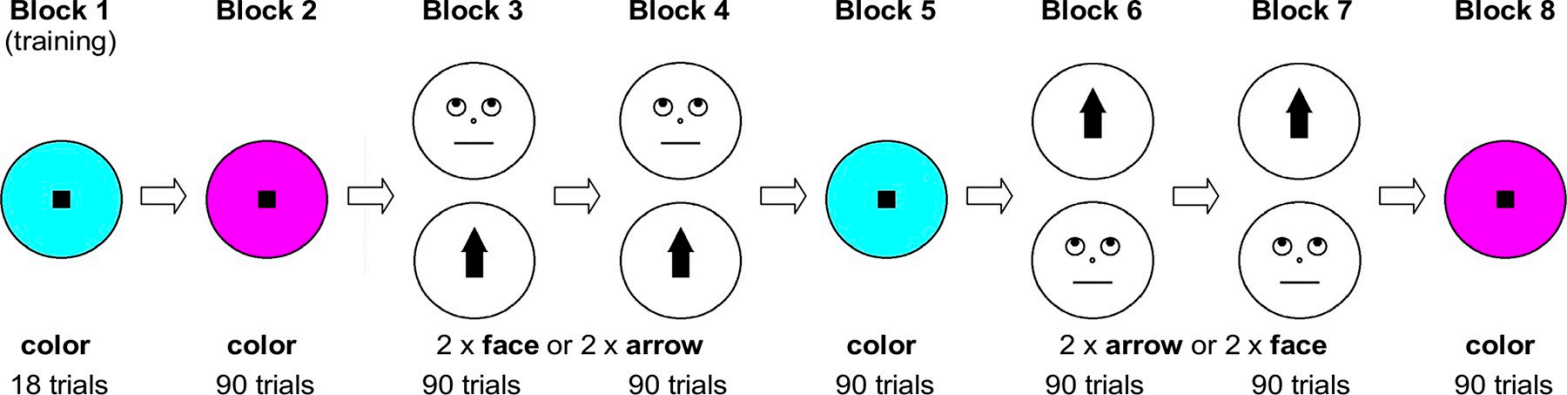
Sequence of blocks in Experiment 2. A training block (cue type: color) was followed by 7 experimental blocks. In Blocks 2, 5 and 8, the cue type was color; in half of the participants face cues were used in Block 2 and Block 3 and arrow cues in Block 6 and Block 7, for the other half face blocks and arrow blocks were reversed.

The two blocks of faces and arrows in each case were presented one after the other. Whether participants saw faces in blocks 3 and 4 and arrows in blocks 6 and 7 or the other way round (Fig 4).

Because there was only one axis of cue and target directions, button use differed from Experiment 1. Instead of using one hand, participants pressed the left or right button of the Button Box with the left and right index finger, respectively.

#### Analyses

As measure of accuracy, we used the proportion of correct responses in the trials of the respective condition. Note that we used the proportion rather than the absolute number of correct trials, as there were three color blocks per participant, but only two arrow and face blocks each. We used a 3 × 2 rmANOVA to assess a possible dependency of accuracy on the factors cue type (arrow, face, color) and cueing (cued, neutral).

As for Experiment 1, we used RTs of correct trials to compute the CEs. Note that the CE here only refers to one cueing condition (the sign is defined such that positive a CE implies an advantage in the “valid” direction, i.e., contrary to direction of the gaze or the arrow). To test for a general effect of cue type (color, face, arrow) on CE, we performed a 3 × 1 rmANOVA, and follow-up t-tests for each pair of cue types as appropriate.

While these parametric tests assess whether the mean CE across participants depended on cue type, we also asked whether a majority of participants showed a larger or a smaller CE for the arrow cues as compared to face cues (irrespective of the size of the individual CE difference); to assess this statistically, we applied a chi-square test.

We further tested whether there were any training effects that led to a difference in CE over the course of the experiment. To this end, we performed separate analyses for each cue type, using a 3 × 1 rmANOVA with the factor block (levels: 2^nd^, 5^th^, 8^th^) for the color-cue blocks. For arrows and faces, we compared the first and second blocks in which the cue type was presented with paired t-⁠tests. For these analyses, the CEs were calculated separately for the respective blocks.

### Results

#### Reaction times

As a first step, we analyzed whether each individual’s reaction time varied over the course of the experiment after the initial 18-trial training block had been completed. To this end, we used the color-cueing trials, which in all participants were consistently placed as block 2, 5 and 8. All but one participant were consistent over time with differences between median reaction times of subsequent color-cueing blocks below 200 ms (range: 2 ms– 184 ms). In Experiment 2b, however, we identified one participant who showed substantially more inconsistency with a slowing of 266 ms from block 5 to block 8. Incidentally, this participant also had the second highest reaction times overall and–of those participants with usable eye-tracking data–the most abundance of late (>450ms) first saccades. Hence, we excluded this participant from all further analyses, leaving N = 23 for Experiment 2b and N = 39 for Experiment 2.

#### Accuracy

Error analysis revealed an accuracy (% correct responses) of 95.7% for Experiment 2 (Experiment 2a: 94.9%, Experiment 2b: 96.3%). A 3 × 2 rmANOVA indicated no differences in accuracy for the factors cue type (arrow, face, color) and cueing (cued, neutral) and no significant interaction between these factors (all *p*s > .8). Only when the data from Experiments 2a and 2b were analyzed together, a significant difference between cued and neutral trials was found (Table 1). Comparisons of RTs between correct and incorrect responses showed significantly smaller RTs for incorrect trials (*p* < .001, Table 1).

**Table 1:** Results of Experiment 2a, its replication Experiment 2b and, of the combined samples, Experiment 2 (significant values in bold font). Note that for consistency, we report follow-up tests on cueType also for Experiment 2a (italics), although the 3 × 1 rmANOVA has only a trend to a main effect of this 3-level factor.

	Experiment 2a	Experiment 2b	Experiment 2 (2a and 2b combined)
**Sample size**	N = 16	N = 23	N = 39
**Accuracy**			
3 × 2 rmANOVA			
Cue Type	F(2, 30) = 1.02, p = .35	F(2, 44) = 2.34, p = .13	F(2, 76) = 2.92, p = .08
Cueing	F(2, 15) = 3.50, p = .08	F(2, 22) = 3.25, p = .09	**F(1, 38) = 6.89, p = .01**
Cue Type: Cueing	F(2, 30) = 1.82, p = .20	F(2, 44) = 0.82, p = .45	F(2, 76) = 2.22, p = .12
t-test: RT correct vs. incorrect	**t(15) = 10.19, p < .001**	**t(22) = 4.85, p < .001**	**t(38) = 8.27, p < .001**
**Cueing Effects** (CE)			
Cueing Effects			
** **t-test: arrow	**t(15) = 3.05, p = .01**	**t(22) = 3.02, p = .01**	**t(38) = 4.29, p < .001**
** **t-test: face	t(15) = 1.77, p = .10	t(22) = 1.27, p = .22	**t(38) = 2.18, p = .04**
** **t-test: color	**t(15) = 2.74, p = .02**	**t(22) = 6.04, p < .001**	**t(38) = 5.76, p < .001**
Cue Type Differences			
** **3 × 1 rmANOVA: Cue Type	F(2, 30) = 3.26, p = .052	**F(2, 44) = 5.97, p = .005**	**F(2, 76) = 8.63, p < .001**
** **paired t-test: face vs. arrow	***t(15) = 2*.*43*, *p =* .*03***	t(22) = 1.70, p = .10	**t(38) = 2.63, p = .01**
** **paired t-test: face vs. color	*t(15) = 1*.*99*, *p =* .*06*	**t(22) = 4.53, p < .001**	**t(38) = 4.70, p < .001**
** **paired t-test: arrow vs. color	*t(15) = 0*.*14*, *p =* .*89*	t(22) = 1.48, p = .15	t(38) = 1.40, p = .17
Number of participants			
** **chi-square test: relation of CE arrow > CE face	**X**^**2**^ **(1, N = 16) = 6.25,** **p = .01**	X^2^ (1, N = 23) = 3.52, p = .06	**X**^**2**^ **(1, N = 39) = 9.26,** **p = .002**
Differences of repeated Conditions			
** **3 × 1 rmANOVA: block 2, 5, 8	F(2, 30) = 2.27, p = .12	F(2, 44) = 0.30, p = .74	F(2, 76) = 1.81, p = .17
** **paired t-test: arrow block A vs. B	t(15) = 0.38, p = .71	t(22) = 1.43, p = .17	t(38) = 1.29, p = .21
** **paired t-test: face block A vs. B	t(15) = -0.13, p = .90	t(22) = 0.44, p = .66	t(38) = 0.25, p = .80

#### Cueing effects

We first analyzed CEs separately for each cue type. For color and arrows we found significant positive CEs in the original experiment (Experiment 2a), the replication (Experiment 2b) and their combination (Experiment 2), with all *p*s < .02 (Table 1). CEs for faces were more brittle, we did not find them for the isolated Experiments 2a and 2b (all *p*s > .1), but only for the combination (Experiment 2, *p* = .04, Table 1). This is an indication that counter-predictive cueing is more robust for arrows than for faces.

**Fig 5:**
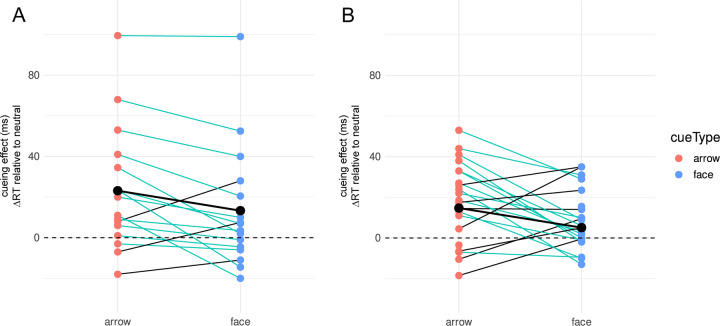
Cueing effects of Experiment 2a and 2b. Red and Blue: Individual cueing effects (CE) based on medians of each of the 16 (Experiment 2a) or 23 (Experiment 2b) participants for arrow and face. *Black markers and bold black line*: means of the CEs. *Thin cyan lines*: participants who showed larger CE for arrows than for faces; *thin black lines*: participants with larger CEs for faces than for arrows. Note that in Experiment 2, positive CE values show a reaction time advantage for the predicted direction that is opposite to the stimulus’ inherent direction for arrows and faces.

To statistically quantify whether there was indeed an observable difference between the cue types, we first included all three cue types (color, face, arrow). We found a trend to a main effect for Experiment 2a (*p* = .052, see Table 1 for all test statistics), an effect we replicated in Experiment 2b (*p* = .005). Follow-up t-tests showed no difference between color and arrow–neither in the original experiment, nor in the replication nor in their combination (all *p*s > .15). Face cues had already tended to a smaller CE than color cues in Experiment 2a (*p* = .06), a tendency confirmed in Experiment 2b (*p* < .001) and in the combined sample (*p* < .001). For the difference of interest, between faces and arrows, the results were somewhat mixed. While we found a difference in the original Experiment 2a (*p* = .03), only a trend was observed in Experiment 2b (*p* = .10) and the combined data of Experiment 2 showed a significant difference (*p* = .01), in all cases with smaller counter-predictive cueing effects for faces than for arrows. Together, these analyses show that counter-predictive cueing with arrows is comparable to predictive cueing with colors. In turn, counter-predictive cueing with faces is less robust than counter-predictive cueing with arrows (and than predictive cueing with colors), although we could establish differences between arrows and faces only with comparably large sample sizes. One potential reason, why comparably large sample sizes were required to establish a difference between arrows and faces, despite the robust CEs for the former and the brittle CE for the latter, is in the large inter-individual variability in CEs and CE differences (Fig 5). Besides asking whether there is a difference between arrow and face CE *on average*, we therefore also asked whether a majority of participants had a larger CE for one particular cue type. For the full Experiment 2 sample, we indeed found that significantly more participants had a larger CE for arrows than for faces (29/39; cyan lines in Fig 5) than vice versa (10/39; thin black lines in Fig 5). This also held for the Experiment 2a subsample (13/16 vs. 3/16) and the same trend was observed for the Experiment 2b subsample (16/23 vs. 7/23; Fig 5 and chi-⁠square results in Table 1).

The main purpose of the additional color condition was to test if there were any training effects that led to a CE difference during the course of the experiment, but there was no effect of block number. For arrows and faces, we compared the first and second blocks in which the cue type was presented: We found no significant CE difference for either the arrow condition or the face condition (see Table 1).

In summary, the behavioral data of Experiment 2 show that arrows can be used as cues against their inherent direction, while this is for most participants not possible or at least harder for gaze cues. As we see no evidence for a change of cueing effects during the experiment, it is unlikely that this difference is due to a lack of training on the time scale of the experiment or that it would be alleviated on a longer, experimentally feasible timescale.

#### Eye movements

**Fig 6:**
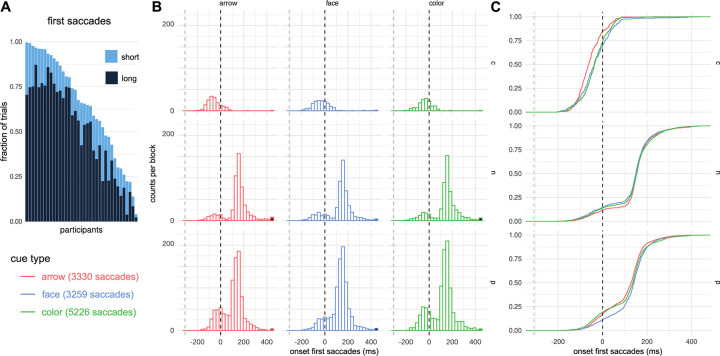
First saccades. A) Fraction of trials in which each participant performed a saccade, participants sorted by the fraction of saccades, long (>2dva) and short (< = 2dva) saccades stacked. B) Histogram of Saccade latencies separated by condition. Only experimental blocks (Blocks 2 through 8) and saccades of more than 2dva were considered. To achieve comparable axes, the absolute number of saccades divided by the number of blocks of the respective cue type is plotted (3 color blocks and 2 arrow and face blocks). C) Cumulative density function for the data of panel B. Gray dashed line shows cue onset and black dashed line shows target onset. Saccade categories: c: saccades against (counter) the predicted direction (following arrow and face, but against color cue), n: saccades of trials with neutral cues, p: saccades in the predicted direction. Saccade onset in ms relative to target onset.

In Experiment 2, participants were free to move their eyes at any point in the trial, but eye movements were not required to perform the task. As the gaze analysis was exploratory and not part of the replication of Experiment 1, the results refer to the whole of Experiment 2 (2a and 2b combined). Due to missing eye-tracking data, we excluded two further participants, such that the training and experimental blocks of *N* = 37 individuals were included in the eye-movement analysis. We focused on the first saccade in the trial that occurred at least 100 ms after cue onset (as earlier saccades are unlikely driven by the cue) and considered its vertical component only. In 35% of trials no such saccade was detected, in 14% of the trials saccades were made to positions less than 2 dva above or below central fixation and in 51% of the trials a saccade of more than 2 dva in the vertical direction was detected. We restricted our qualitative analysis to the latter saccades and aggregated across all observers, but note that these saccades were distributed neither equally across individuals (Fig 6A) nor across conditions (Fig 6B).

We classified saccades according to their direction: saccades in the direction predicted by the cue (p), saccades that go in the opposite (counter) direction (c), which is the stimulus-inherent direction for faces and arrows. Although participants did not need to fixate the target to perform the task, “p”⁠ saccades bring the target closer to the fovea and therefore potentially foster task performance, while “c” saccades move the target away from the fovea, potentially harming task performance. In addition, we considered saccades of all directions in neutral-cue trials (n). The longest RTs (*Mdn* = 523 ms) were found in trials with c-type saccades, the shortest were found for trials with p⁠-⁠type saccades (*Mdn* = 454 ms) with the n-type saccades (i.e., neutral trials with saccades) fell in⁠-⁠between (*Mdn* = 488 ms).

For all cue types, we found similar distributions of the onsets of the first saccades. The differences between the saccade categories were more striking: in category c (Fig 6B, top), only early first saccades occur, while the distribution of the other two categories seems to be composed of two distributions: as with the category c, there were some early saccades that can be interpreted as responding to the cue onset, to which there was a larger set of later first saccades that can be interpreted as responding to the target onset. The distributions for trials in which the first saccades go in the predicted direction (p, Fig 6B, bottom) were very similar to distributions of trials in which there was only a neutral cue (n, Fig 6B, middle).

The empirical cumulative distribution functions (ecdfs) of the first saccades suggest that arrows trigger earlier first saccades in the “wrong” (i.e., inherent) direction (Fig 6B, c) than the faces. Also, later saccades of arrow trials in the “correct” (predicted) direction seem to start earlier than in the face condition (Fig 6B, p). For c-saccades the ecdfs of face and color cues were more similar to each other than either of them was to the ecdfs of arrow cues; conversely, for p-saccades the ecdfs of arrow and color were closer to each other than either was to the ecdf of face cues. The ecdfs of saccades following neutral cues showed no obvious differences. Note that we deliberately restrict ourselves to a qualitative description of the eye-movement data, as the experiment was not primarily designed for these analyses, which would–for example–require to enforce saccades either by instruction or by rendering the targets so small that foveal vision is required for identification. Nonetheless, we consider this description instructive, in particular as allowing participants to move their eyes may constitute a more natural viewing setting than instructing or enforcing fixation.

## Discussion

We investigated attention allocation using centrally presented cues. In Experiment 1, we replicated the findings of Friesen and Kingstone for their 300ms SOA identification task [9]. Specifically, we obtained the same overall result for CEs evoked by faces, with a significant RT advantage for cued targets compared to both uncued targets and trials with neutral cues, with no additional attentional cost for the uncued condition. Beyond replicating the original study, we examined CEs on the vertical axis and with arrows as cues. We found no difference between the axes and effects of arrows were indistinguishable from those of faces. The similarity between the axes provides more flexibility for cuing experiments, in which target locations so far have often been restricted to the horizontal axis, and also widens options for practical applications of gaze cueing. The general qualitative similarity of arrows and gaze cues is consistent with recent findings [13]. The close quantitative match between our arrows and faces in Experiment 1 in addition allowed us to test whether there are non-trivial differences between arrows and faces when they are counter-predictive, that is, when their inherent meaning has to be overridden to make use of their validity.

In each of Experiments 2a and 2b, we indeed found significant RT advantages for counter-predictive arrows towards the predicted (valid, but uncued) side. There also was some counter-predictive cueing effect for faces, but the power of the combined sample was needed for this effect to reach significance. This suggests that the cueing effect is less robust for faces than for arrows. RT advantages do not seem to require extensive learning, as we see no indication that CE change over the course of the experiment, neither for the cues with inherent meaning (arrows and faces) nor for color cues that served as control for this purpose. Overall, CE was also significantly greater for arrows than for faces. However, this difference was not significant for Experiment 2b and there was substantial inter-individual variability in CE and CE differences. Although overall significantly more participants showed greater CE for arrows than for faces, the sample of Experiment 2b contained several participants (7/23) who showed comparably large differences in the opposite direction (thin black lines in Fig 5B). From our experiments with a single session per participant, it remains open whether the observed inter-individual variability in the ability to override a certain type of cue and in CE in general is indeed the manifestation of a general, persistent individual trait or merely reflects the state of the individual at the time of the experiment. Assessing whether susceptibility to cues and the ability to override them are stable across sessions in the same individual may therefore be an interesting issue for further research. In the context of human-robot interaction, this would become particularly relevant, if the robotic interaction partner is not encountered incidentally, but part of a continuous and repeated interaction with the same human individual.

When comparing Experiment 1 to Experiment 2, it is remarkable that cues that are apparently very similar when unpredictive–faces and arrows show very similar cueing effects in Experiment 1 –have different effects when they become counter-predictive, i.e., are 100% valid but contrary to their inherent meaning. Except for the neutral cues, cues in Experiment 2 were predictive in 100% of the trials. Hence, from an informational point of view, shifting attention based on the directional cues would be helpful to solve the task in all non-neutral trials. Our results suggest that this shift is more easily accomplished against the inherent directional meaning of an arrow than against the direction of gaze. For both–arrows and faces–in Experiment 2, participants must associate a new and unusual directional mapping and therefore inhibit processes that are reflexive to the inherent direction implicated by the cue. Apparently, this inhibition is achieved more readily for arrows than for faces. This is in line with data based on cue-target congruency that also suggests that gaze and arrow cues can elicit reflexive shifts of spatial attention, but that the attentional effect elicited by gaze direction is more reflexive [33] and may therefore be harder to override. However, these differences do not necessarily argue against the existence of shared processes for arrows and gaze cues. It is likely that, even in the context of counter-predictive cueing, shared processes are responsible for the occurrence of cueing effects of both cue types. The differences found in this study, nevertheless, also contribute to the growing evidence that there are also specific processes in addition to these shared processes [13]. The occurrence of such specific processes seems to be context-dependent, such that differences like those found in our study argue against gaze cueing being a purely automatic process [34]. In our design, participants made only few errors, such that the connection between reflexive and inhibitory processes can hardly be inferred from behavioral data. However, we deliberately allowed participants to move their eyes during Experiment 2. Besides avoiding the need to actively suppress eye movements, which in itself can interfere with the task, this allowed us to analyze the directional distribution of the initial saccade with respect to target location and cue direction. The logic of this analysis to dissociate reflexive from cognitively controlled effects follows the ideas of so-called countermanding and anti-saccade tasks. In countermanding, participants are asked to make saccades in a given direction, but on a subset of trials they receive a delayed signal to stop the saccade [35]. It then takes time to inhibit the production of initiated saccades because stop and go processes are active simultaneously. When the stop stimulus appears late, participants make countermanding errors, i.e., the go process reaches an activation threshold before the stop process. Applying this principle, our data suggest that the inhibition process starts quite early, since we see no evidence for errors caused by a process that is reflexive to the cue’s inherent direction. Anti-saccade tasks ([36] for a review) require participants to make a saccade in the opposing direction to a salient target. Conceptually, this is similar to our present task, with the salient exogeneous stimulus being replaced by an overtrained meaning of gaze or arrow, which in fact requires an attentional shift in the opposite direction.

At large, the distribution of saccade data in our study is very similar for the different cue types, suggesting a shared process contributing to the cueing effects. Nonetheless, we find small differences that are of interest with respect to additional, cue-specific processes: There are a couple of early saccades that follow the cue rather than supporting target identification through a gaze shift to the (future) target side. However, in our study, such early saccades against the direction predicted by the cue also occur in the color condition; this suggests that following the (face or arrow) cue’s inherent direction is responsible only for a fraction of these "wrong" saccades. Remarkably, the fraction of early wrong gaze shifts seems to be larger for arrows than for faces. In turn, there were fewer “helpful” early gaze shifts (i.e., early shifts in the direction of the target) for faces than for arrows. Hence–although our experiment had not explicitly been designed to test this–reflexive gaze shifts that are potentially suboptimal for performance in our task (because they follow the inherent direction of the cue) were more abundant for arrows than for faces, but “helpful” gaze shifts (in the direction of the target) also were earlier for arrows than for faces. If true, this would again support an early general inhibitory mechanism that (sometimes unsuccessfully) prevents following a gaze cue, and a specific re-association of the cue towards the opposite direction may be more prevalent for arrows. This is consistent with saccades in face cue trials having a somewhat higher latency than for arrow- and color-cue trials. This difference is conceptually also in line with the observation that in natural scenes gaze towards faces is somewhat harder to avoid than gaze to symbols (text in their case, arrows in our case), although both classes are far harder to avoid than everyday objects [37]. To obtain more eye-movement data consistently across observers and conditions, which would allow for a more quantitative analysis, future experiments could use an explicit saccade task (like, e.g., [14]) and ask participants to saccade against the arrow or the gaze. Alternatively, keeping the present setting, the identification target could be rendered sufficiently small that its foveation is required to accomplish the task. In any case, the notion of an early, general inhibition (for faces) and tendency for re-association (for arrows) are in line with our behavioral data, as it would explain the differences between arrows and faces as well as the absence of any noticeable learning effects on the CE, provided both are set by task instructions (or acquired in very few trials). For the detailed investigation of earlier and later processes, small-step variations of the SOA might be a promising approach. While we chose our cue-target SOA of 300 ms based on the results of Friesen and Kingstone [9], different SOAs could possibly lead to larger effects [13]: at shorter SOAs, there is evidence for reflexive orientation of attention in the cue-inherent direction, while longer SOAs may allow a more controlled allocation of attention, leading to reaction time advantages in counter-predictive tasks [see ref. 13 for an overview]. An SOA of 300 ms would then fall in a kind of “transitional” range, before more controlled processes become dominant over reflexive processes. As there are indications that the ability to control volitional inhibition depends on individual factors [22], it would also be helpful to investigate this in studies designed specifically for this question, explicitly assessing interindividual variability.

Simplified schematic faces in neutral backgrounds, as used here and in [9], have proven most useful to reveal fundamentals of cueing. For research towards applications of gaze cueing, it will be most valuable to determine the extent to which the thus obtained results transfer to more complex contexts and settings as well as to assess the role of the cue’s complexity. For example, in a setting with actual faces and complex backgrounds, observers’ precision in estimating gaze direction is worse in the vertical than in the horizontal [29], which in turn may affect the precision and efficacy of gaze cues. Regarding the complexity of the cue as such, McKay and colleagues found stronger gaze cueing for real faces in direct comparison to schematic faces [16], but this small effect did not persist in the multiple moderator model in which the effects of direct gaze before the cue, offset timing of the gaze cue and target, and task type were controlled. The complexity of the environment in which we typically learn these cues may also contribute to the differences between faces and arrows in Experiment 2. With the possible exception of deliberate deception [38]–a sender’s gaze is directed to the target and we observe such gaze-target contingencies from earliest infancy [19]. This mapping is unavoidable because the eyes are not primarily indicators of attentional orientation for the receiver (person who receives the gaze) of a gaze cue, but the sender’s (person who sends the gaze) visual sensors. In contrast, the meaning of arrows is probably more task and context dependent. To provide simple everyday examples: arrows indicating that a lane continues straight ahead typically point downward on US American highway signs, whereas they point upward on German highway signs; an arrow key pointing to the right will typically move the cursor to the right, but in most viewers move the content to the left. If a counter-predictive mapping requires reflexive attentional shifts to be volitionally modulated [22], this may be more difficult for cues that appear with a consistent mapping across contexts. In this view, the relative flexibility of arrows’ directional interpretation might make it easier to override them than gaze cues, whose directional interpretation is stable across contexts.

Together our data suggest that gaze is overtrained in a different way than arrows. Applied to human-machine interaction, gaze and arrows may therefore be useful in distinct and complementary roles. This is exemplified in the following scenario: a robot is designed to indicate its imminent movement direction either by an arrow or by a gaze display. A human approaching the robot then has to evade the robot’s path by directing their attention *against* the direction of this cue (this assumes familiarity with the robot and ignores the real-life challenge to decide whether another agent indicates its own intended direction of movement or signals the direction it shall be passed). In the light of our results, an arrow would be more appropriate than a gaze display if response efficiency, i.e., the speed of the evasive movement against the cued direction, is to be optimized. In the context of this scenario, our results are consistent with those of Angelopoulos and colleagues [24], who showed that deictic gesture cues can outperform gaze cues in communicating intended navigational behavior. However, when a robot is approaching a human directly, making eye contact with the human is perceived as more comfortable than looking in the direction of its path [39]. Thus, in such situations, the use of gaze cues would be more appropriate to establish a sense of human-robot engagement and comfort during the interaction, because looking toward the human interaction partner is perceived as information that the robot is attending to the human.

For such applied interaction scenarios, one needs to acknowledge that there might not be strictly distinct categories of cues in terms of the “reflexivity” by which humans react to a cue, rather cues types can be considered as part of a "range" [33] or a continuous space. This view may be particularly useful in human-machine interaction, where the cueing display (arrow, faces, robot faces, pointers, etc.) can be designed flexibly and is not bound to sensory constraints of the sender (unlike in the case of human gaze, where the sender’s gaze direction primarily subserves a sensory function). The strength of cueing paradigms can be used to investigate a particular effect with matched stimuli–as the overriding of cues that are equally effective when unpredictive in our Experiment 2 –or to consider the attentional shifts of a particular gaze display in the development process for practice (e.g., [8]). In summary, the type of experiment conducted in the present study, although designed to assess the effect of simple cues (schematic faces, arrows, colors) in a comparably simple identification task, can provide valuable insights also for the design of human-machine interaction and therefore for the smooth interplay of humans and embodied digital technologies, which we expect to play an increasingly visible role in the foreseeable future.
